# The effect of cortisol in early pregnancy on postpartum depressive symptoms

**DOI:** 10.1038/s41598-025-88772-0

**Published:** 2025-04-16

**Authors:** Małgorzata Sobol, Inna Hryhorchuk, Elżbieta Plucińska, Paulina Szczepaniak, Agata Błachnio, Janusz Stasiniewicz, Marek K. Sobol

**Affiliations:** 1 Department of Psychology, University of Warsaw, Ul. Stawki 5/7, 00-183 Warsaw, Poland; 2 Żywiec Hospital, Ul. Pola Lisickich 80, 34-300 Żywiec, Poland; 3 John Paul II Catholic University of Lublin, Al. Raclawickie 14, 20-950 Lublin, Poland; 4 Hospital Center Châlons-En-Champagne, Châlons-En-Champagne, France

**Keywords:** Pregnancy, Postpartum depression, Postpartum stress, Time perspective, Hair cortisol concentration, Physiology, Psychology, Endocrinology, Risk factors

## Abstract

The first months after childbirth are a tremendous challenge for women and, consequently, a time when women’s mental health problems often arise. Knowledge of the prenatal predictors of these problems is of fundamental importance in preventing them. This study aimed to test whether first trimester hair cortisol influenced maternal postpartum depressive symptoms. The women (*N* = 75) were tested twice: in the first trimester of pregnancy and within three months after giving birth. In the first trimester, they had hair samples taken and were examined using a sociodemographic survey and questionnaires: the Edinburgh Postnatal Depression Scale (EPDS), the Perceived Stress Scale (PSS-10), and the Zimbardo Time Perspective Inventory. After delivery, women completed a survey about the course of delivery and their child’s health, EPDS, and PSS-10. Low hair cortisol concentration in the first trimester was a predictor of a high level of postpartum depressive symptoms. This relationship was mediated by fatalistic time perspective. The results suggest that low hair cortisol concentration in the first trimester of pregnancy indicates a high probability of postpartum depression, and that low levels of cortisol may be associated with passivity, a sense of lack of control, and helplessness.

## Introduction

During the postpartum period, women are particularly vulnerable to mental health problems such as postpartum depression^1^. The incidence of postpartum depression ranges from 3.9% to as high as 69.9%, depending on the country^2^. This disorder has a negative impact on women’s physical and mental health, and in extreme cases it may even lead to suicide or infanticide^3^. The first step in the prevention process is to identify the women in early pregnancy who are in the risk group for developing postpartum depression^4^. Research shows that a woman’s mental state and behavior in the first trimester are related to her condition after giving birth^5,6^.

To date, much research has been conducted on behavioral and psychological predictors of postpartum depression, but still very little is known about biological ones^7^. One of the strongest and at the same time easily available biological markers of negative affect is hair cortisol concentration^8^. Accordingly, the present study aimed to examine the effect of hair cortisol concentration in the first trimester on women’s postpartum depressive symptoms. Additionally, the role of time perspective^9^ in explaining this relationship was estimated. The study was conducted in Poland.

Cortisol is an adrenal hormone, the final product of the hypothalamic–pituitary–adrenal (HPA) axis^10^, and plays an important role in stress control, energy regulation, and the normal functioning of the immune and anti-inflammatory systems. Changes in the HPA axis can be triggered by stressful experiences and by past or present physical-economic conditions^11^. One of the types of biological materials used for assessing long-term cortisol secretion are hair samples. On average, hair grows about 1 cm per month, so it is assumed that the cortisol content in 1 cm of hair reflects the level of cortisol in the last month^12^.

One of the most well-known phenomena in biological psychiatry is the association of depression with elevated cortisol levels in adults^13^. However, some research has shown that high cortisol levels are not always bad and low cortisol levels are not always good^14^. The complex, adaptive importance of high cortisol levels in coping with the challenges of everyday life has been emphasized^14,15^. In turn, low cortisol levels was associated with cumulative risk exposure to stress in adults^16,17^. Research revealed a relationship between low cortisol levels and perceiving challenges as insurmountable and long-term^18,19^. Chronic postpartum depression was more often associated with low cortisol levels, while transient postpartum depression with high cortisol levels^20^.

Understanding the relationship between the activation of the HPA axis during pregnancy and postpartum health still remains a challenge for researchers^7^. Previous studies yielded inconsistent results. A high level of postpartum depression symptoms was associated with a low level of salivary cortisol in the first trimester among low-income women from the USA^21^. In contrast, Caparros-Gonzalez et al.^7^ found that women from Spain, with a high intensity of postpartum depression had higher hair cortisol concentration in the first trimester than women with a lower intensity of postpartum depression.

When comparing the results of research on the associations of cortisol levels with depression, it is advisable to consider cultural and social factors^22^. In previous studies, overall low cortisol levels usually co-occurred with high levels of self-reported stress, depression and difficult sociodemographic conditions^23–25^. Moreover, research showed that hair cortisol concentration in the first trimester varied, depending on the country and the woman’s sociodemographic situation. For example, hair cortisol concentration was equal to 5 pg/mg in a group of women from Peru, where more than half of whom were unemployed and had a difficult financial situation^25^. Hair cortisol concentration was 25 pg/mg in a group of women from the USA, in which only one woman was unemployment^26^. Research results from the last few years indicate a generally low level of cortisol at the beginning of pregnancy among Polish women^27,28^. Low cortisol levels among pregnant women in Poland may be explained by chronic stress related to restrictive laws regarding the possibility of terminating pregnancy. It can also be interpreted in the context of the concept of generational trauma related to the war experiences of previous generations^28–30^. That is why we formulated the following hypothesis:

Hypothesis 1

A lower level of hair cortisol concentration in the first trimester is associated with a higher level of postpartum depressive symptoms.

Furthermore, we hypothesized that the association between low cortisol and postpartum depression would be explained by a time perspective. Time perspective is a tendency to focus on the past, present or future, connected with a positive or negative evaluation of time^9^. Previous studies found that fatalistic and past negative perspectives have particularly negative consequences^9,31,32^. Fatalistic perspective is passivity and the belief that one has no influence on events, perceiving the future as predetermined, and a sense of helplessness. Past negative perspective is a tendency to frequently return to negative memories from the past and to evaluate past events in a negative way^9^. Previous research indicates that low hair cortisol concentration often coexists with the experience of chronic stress and unfavorable socioeconomic factors^24,25^. Such a life situation is usually associated with a feeling of powerlessness and hopelessness, a sense of lack of control over life, and negative past experiences^9^. On this basis, we formulated the following hypothesis:

Hypothesis 2

The association of hair cortisol concentration in the first trimester with postpartum depressive symptoms is explained by present fatalistic and past negative time perspectives.

## Method

### Participants

Participants were pregnant women (*N* = 78), patients of an outpatient gynecological clinic and a private gynecological office in Poland. Women at gestational week 4–13 were recruited during gynecological visits. Psychiatric illness and multiple pregnancy were exclusion criteria. The research was conducted between May 2021 and April 2024.

### Procedure

The assessments were performed in the first trimester of pregnancy and during three months after delivery. In these two stages, women received e-mails with links to online questionnaires. Moreover, two strands of hair, cut close to the scalp, were collected during the first trimester. Each patient received a monetary remuneration of EUR 137. All research was performed in accordance with the principles of the Declaration of Helsinki. Informed consent was obtained from all participants. The research protocol was approved by the University of Wrocław Ethics Committee. Study hypotheses, methods and procedure were pre-registere using the Open Science Framework repository (https://osf.io/4aysp/).

### Measures

Sociodemographic and medical surveys were used and gynecological pregnancy evaluation was performed, which covered the following: cervix examination, fetus development assessment, examination for threatened miscarriage, blood glucose level test, TSH test, the course of delivery, and the newborn health conditions, weigh, and length.

The Edinburgh Postnatal Depression Scale (EPDS)^33^ was used to assess perinatal depressive symptoms in the past two weeks, including feelings of sadness, blaming oneself, and unwarranted anxiety. The measure consists of 10 items (e.g., “I have been so unhappy that I have had difficulty sleeping”; “I have felt sad or miserable”), rated on a 5-point Likert-type scale (the range scores from 0 to 30; ). In this study, Cronbach’s α was 0.86.

The Present Fatalistic (PF, 9 items, the range scores from 9 to 45) and Past Negative (PN, 10 items, the range scores from 10 to 50) scales of the Zimbardo Time Perspective Inventory^9^ were used to measure passivity stemming from the belief that life is determined by fate (example items: “Fate determines much in my life”; “My life path is controlled by forces I cannot influence”) and the focus on the negatively evaluated past (e.g., “I think about the bad things that have happened to me in the past”; “It is hard for me to forget unpleasant images of my youth”), respectively. Each item is rated on a 5-point Likert-type scale. In this study, Cronbach’s α was 0.75 for the Present Fatalistic scale and 0.85 for the Past Negative scale.

The Perceived Stress Scale (PSS-10)^34^ was used to measure subjectively perceived stress, defined as feelings of being upset, stressed, and unable to cope in the past month. The measure consists of 10 items (e.g., “In the last month, how often have you felt nervous and stressed?”; “In the last month, how often have you felt difficulties were piling up so high that you could not overcome them?”) rated on a 5-point Likert-type scale (the range scores from 0 to 40). In this study, Cronbach’s α was 0.80.

Hair cortisol concentration was measured in hair strands (3 mm in diameter), cut as close as possible to the scalp from a posterior vertex position^35^. We stored hair samples in a dry place, wrapped in aluminum foil. Three-centimeter segments (5–15 mg) were analyzed, covering the past 3 months^35^. Hair samples were washed in 3 mL isopropanol for 3 min (two times). Steroid hormones were extracted from 7.5 mg of whole, non-pulverized hair using 1.8 mL methanol for 18 h at room temperature, then 1.6 mL of the clear supernatant was transferred into a new 2 mL tube. The alcohol was evaporated at 50 °C under a constant stream of nitrogen and reconstituted with 225 µL double-distilled water and 20 µL of a mixture of cortisol-d4. 50 µL of this extract were injected into a Shimadzu HPLC-tandem mass spectrometry system (Shimadzu, Canby, Oregon) coupled to an SCIEX API 6500 turbo-ion-spray triple quadrupole tandem mass spectrometer (SCIEX, Foster City, California). The lower limits of quantification (LOQ) for this analysis were below 0.3 pg/mg. The analyses were conducted by the Biochemical Laboratory, Technische Universitat Dresden, Germany. The hair cortisol concentration value for each of the two hair strands were estimated, and the average hair cortisol concentration index was calculated.

### Statistical analyses

Power analysis^36^ for *t*-test, linear multiple regression fixed model, single regression coefficient, one tail, effect size ƒ^2^ = 0.15, with the alpha level set to 0.05 and power set to 0.95, was used to determine sample size. We relied on the average effect size estimate in individual differences research (*r* ≈ 0.20)^37^. The power analysis indicated 74 women as the required sample size. A larger sample was recruited to allow for attrition. We used the SPSS 27 statistical package to perform the analyses.

We conducted correlation analyses to determine the associations between the variables. A multiple hierarchical regression analysis was performed to identify the variables that contributed most to postpartum depressive symptoms. Moreover, we performed mediation analyses to determine the indirect effect of prenatal hair cortisol concentration on postpartum depressive symptoms through time perspective^38^. We operationalized the indirect effect as the product of the regression path between prenatal hair cortisol concentration and time perspective and the path between hair cortisol concentration and postpartum depressive symptoms. Bootstrapped mediation analyses in PROCESS 3.5^39^ with bootstrapped samples were used to test the direct and indirect effects. A 95% bootstrap confidence interval for the indirect effect of hair cortisol concentration was generated. In all analyses, perceived stress level was included as a controlled variable. The results were significant at *p* < 0.05.

## Results

### Participants

Of the 78 women recruited, 75 women (age: 21–40 years) successfully completed the second assessment, within 13 weeks after giving birth. Three women had miscarriages. All women in the sample were Polish. Women who dropped out of the study did not differ in hair cortisol levels, stress levels, time perspectives, and perinatal depression from women who remained in the study. The participants had a university degree (59%), secondary education (39%), or elementary education (2%); they lived in a village (25%), a medium-sized town (27%), or a big city (48%). They reported their material situation as very good (12%), good (64%), or average (24%). For 61 women, it was their first childbirth. The participants’ medical characteristics are presented in Table 1.

**Table 1:** Characteristics of medical variables.

Variable	Value	
	Yes	No
Miscarriages in the past	20 (27%)	55 (73%)
History of mood disorders	4 (5%)	71 (95%)
Cases of postpartum depression in mother or sister	5 (7%)	70 (93%)
The presence of a chronic disease (hypothyroidism, anemia)	18 (24%)	57 (76%)
Normal fetal development in the first trimester	73 (97%)	2 (3%)
Symptoms of threatened miscarriage in the first trimester	7 (9%)	68 (91%)
Blood glucose level in the first trimester < 92	71 (95%)	4 (5%)
Blood thyrotropic hormone level in the first trimester < 2.5	69 (92%)	6 (8%)
Premature birth (< 37 week)	74 (99%)	1 (1%)
Caesarean section	43 (57%)	32 (43%)

### Descriptive statistics and correlation analysis

Descriptive statistics and correlations between the variables are presented in Table 2 (first trimester) and Table 3 (postpartum). Because cortisol values were not normally distributed, we applied a logarithmic transformation. The correlations between medical characteristics and the remaining variables are presented in Appendix 1. Twenty five women (33%) had an EPDS score above 12 during postpartum, which is considered to suggest a high level of postpartum depression symptoms see^31^.

**Table 2:** Means, standard deviations, and correlations between study variables in the first trimester.

	1	2	3	4	5	6	7	8	9
1. age	–								
2. education^	0.43***	–							
3. financial situation^^	0.16	0.25*	–						
4. place of living^^^	0.38***	0.27*	0.09	–					
5. HCC I	0.04	− 0.21	0.04	0.06	–				
6. EPDS I	0.20	0.23	0.08	0.13	− 0.32**	–			
7. PSS-10 I	0.16	0.16	0.01	0.03	− 0.18	0.70***	–		
8. PF I	− 0.15	− 0.01	− 0.21	− 0.16	− 0.21	0.32**	0.28*	–	
9. PN I	− 0.07	0.06	− 0.21	− 0.09	− 0.25*	0.53***	0.52***	0.57***	–
10. parity	0.24*	0.01	0.16	0.23*	− 0.02	− 0.04	− 0.01	0.09	0.07
M	31.21	–	–	–	4.16^^^^	8.09	19.12	2.6	2.75
SD	4.34	–	–	–	5.92	5.06	5.90	0.62	0.75

*p < 0.05; **p < 0.01; ***p < 0.001; HCC I: hair cortisol concentration in the first trimester; EPDS I: Edinburgh Postnatal Depression Scale in the first trimester; PSS-10 I: Perceived Stress Scale in the first trimester; PF I: Present Fatalistic scale in the first trimester; PN I: Past Negative scale in the first trimester; ^ 1 – primary, 2 – secondary, 3 – higher education; ^^ 1 – average, 2 – good, 3 – very good; ^^^ village, small town, big city; ^^^^ pg/mg.

**Table 3:** Means, standard deviations and correlations between study variables postpartum.

	1	2	3	4	5	6	7	8	9
1. Age	–								
2. Education^	0.43***	–							
3. Financial situation^^	0.16	0.25*	–						
4. Place of living^^^	0.38***	0.27*	0.09	–					
5. Child’s height	− 0.10	− 0.15	− 0.10	− 0.21	−				
6. Child’s weight	0.04	0.01	0.08	0.02	0.50***	–			
7. Child’s health^^^^	− 0.07	0.05	0.27*	0.16	− 0.12	0.10	–		
8. EPDS II	− 0.16	0.01	0.10	0.09	− 0.09	− 0.11	0.05	–	
9. PSS-10 II	− 0.02	0.18	0.02	0.08	− 0.36**	− 0.09	0.02	0.64***	–
M	32.03	–	–	–	53.9	3393.3	–	8.92	20.57
SD	4.62	–	–	–	3.83	520.52	–	5.92	6.41

*p < .05; **p < .01; ***p < .001; EPDS II: Edinburgh Postnatal Depression Scale during postpartum; PSS-10 II: Perceived Stress Scale during postpartum; ^ 1 – primary, 2 – secondary, 3 – higher education; ^^ 1 – average, 2 – good, 3 – very good; ^^^ village, small town, big city; ^^^^—1 – poor, 2 – moderate, 3 – good, 4 – very good.

In the first trimester, a higher level of depressive symptoms was associated with lower hair cortisol concentration and higher perceived stress. There was no relationship between perceived stress and hair cortisol concentration. Higher levels of both present fatalistic and past negative perspectives were associated with higher depressive symptoms and higher perceived stress (Table 2).

During postpartum, higher depressive symptoms were associated with higher perceived stress. There was an association between higher hair cortisol concentration and lower past negative perspective. Higher levels of perceived stress were associated with lower newborn height. There was also a relationship between place of residence and the child’s health—the larger the place of residence, the healthier the parents reported the child to be (Table 3). Moreover, cases of postpartum depression in mother or sister were associated with higher levels of depressive symptoms and perceived stress in the first trimester. The presence of chronic illness was associated with a higher level of perceived stress during postpartum (Appendix 1).

In regard to correlations of cortisol and psychological variables in the first trimester with postpartum mental health, lower hair cortisol concentration (*r* =  − 0.38, *p* < 0.001), a higher level of prenatal depressive symptoms (*r* = 0.42, *p* < 0.001), higher prenatal stress (*r* = 0.26, *p* < 0.01), higher present fatalistic perspective (*r* = 0.40, *p* < 0.001), and higher past negative perspective (*r* = 0.41, *p* < 0.001) were associated with higher postpartum depressive symptoms.

### Predicting postpartum depressive symptoms

A multiple hierarchical regression analysis was conducted to identify the predictors of postpartum depressive symptoms (Table 4). In the first step, presence of maternal chronic illness and birth length were added, since these variables were associated with postpartum stress in our study. In the second step, we added the scores on depressive symptoms (EPDS) and stress (PSS-10) in the first trimester. After including hair cortisol concentration in the third step, depressive symptoms, stress, and hair cortisol concentration in the first trimester turned out to be predictors of postpartum depressive symptoms. The results showed that higher levels of depressive symptoms and stress and a lower level of hair cortisol concentration in the first trimester were associated with higher levels of postpartum depressive symptoms.

**Table 4:** Regression analyses results for the first trimester predictors of the postpartum depressive symptoms.

Dependent variable	Independent variable	t	Β	R^2^	∆F
EPDS II	Model 1			0.03	
	Step 1				1.90
	Chronic disease	1.81	0.22		
	Newborn growth	− 0.82	− 0.10		
	Model 2			0.16	
	Step1				
	Chronic disease	1.74	0.20		
	Newborn growth	− 0.50	− 0.06		
	Step 2				6.25**
	EPDS I	2.71**	0.42		
	PSS-10 I	− 0.23	− 0.04		
	Final model			0.21	
	Step1				
	Chronic disease	1.38	0.15		
	Newborn growth	− 0.26	− 0.03		
	Step 2				
	EPDS I	2.07*	0.33		
	PSS-10 I	− 0.09	− 0.01		
	Step 3				4.65*
	HCC I	− 2.16*	− 0.25		

*p < .05; **p < .01; ***p < .001; PSS-10 I: Perceived Stress Scale in the first trimester; EPDS I: Edinburgh Postnatal Depression Scale in the first trimester; EPDS II: Edinburgh Postnatal Depression Scale during postpartum; HCC I: hair cortisol concentration in the first trimester;

### The associations of the hair cortisol concentration and time perspective in the first trimester with postpartum depressive symptoms: mediation analyses

**Fig. 1:**
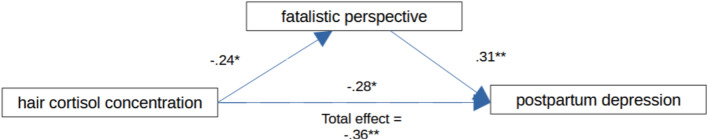
Path analysis for the effect of hair cortisol concentration in the first trimester on the postpartum depressive symptoms through fatalistic time perspective. Note. *p < .05; **p < .01.

We conducted mediation analyses to test time perspective as a mediator in the relationship of hair cortisol concentration in the first trimester to postpartum depressive symptoms (Table 5). As in the case of multiple hierarchical regression analyses, two covariates were included in the analysis: presence of maternal chronic illness and newborn length. A variable is a mediator to the extent that it explains the association of the predictor with the criterion. Based on mediators, it can be concluded how and why the observed relationships between variables occur^40^. If a particular time perspective mediates the association of hair cortisol concentration with postpartum depressive symptoms, this means that hair cortisol concentration in the first trimester influences postpartum depressive symptoms through this time perspective. Two mediation analyses were performed—for present fatalistic time perspective and for past negative time perspective as mediators. The results suggested that fatalistic time perspective mediated the relationship between hair cortisol concentration in the first trimester and postpartum depressive symptoms. In other words, women with lower hair cortisol concentration in the first trimester were more likely to develop postpartum depressive symptoms, and this relationship was explained by fatalistic time perspective (Fig. 1).

**Table 5:** Results of mediation analyses for the relationships of the hair cortisol concentration in the first trimester to the postpartum depressive symptoms with the past negative and fatalistic time perspective in the first trimester as mediators.

Independent variable	mediator	Direct				Indirect				Total			
		B	SE	95% CI		B	SE	95% CI		B	SE	95% CI	
HCC I	PF I	** − 1.81**	**0.73**	** − 3.269**	** − 0.352**	** − 0.48**	**0.30**	** − 1.136**	** − 0.007**	** − 2.30**	**0.74**	** − 3.784**	** − 0.808**
HCC I	PN I	− 1.86	0.73	− 3.316	− 0.408	− 0.43	0.31	− 1.110	0.090	− 2.30	0.74	− 3.784	− 0.808

HCC I: hair cortisol concentration in the first trimester; PN I: Past Negative scale in the first trimester; PF I: Present Fatalistic scale in the first trimester; Statistically significant results are printed in bold type.

## Discussion

The aim of our study was to test whether hair cortisol concentration in the first trimester of pregnancy predicted postpartum depressive symptoms. In addition, we investigated whether time perspective explained this relationship. Our hypotheses were partially supported. As expected (Hypothesis 1), lower hair cortisol concentration in the first trimester was associated with postpartum depressive symptoms when the level of prenatal depressive symptoms, prenatal stress, presence of maternal chronic disease, and birth length were controlled for. Our results are contrary to those obtained by Caparros-Gonzales et al.^7^ in a study of pregnant and postpartum women; they are also contrary to the results of many studies indicating the associations of higher cortisol concentration with a higher level of depressive symptoms^41,42^. However, our results are in line with previous studies suggesting an association of lower salivary cortisol in the first trimester with higher postpartum depression^21^ and lower levels of cortisol in depressed women during posptartum, compared to non-depressed women^20,43^. Our results also correspond to studies that showed associations of hypocortisolism with mental and physical health disorders^44,45^. Moreover, our findings are consistent, to some extent, with the results of studies suggesting associations of a high level of cortisol with positive aspects of psychological functioning, such as experiencing positive and exciting situations^46^, better coping with emotional burden after experiencing stress^15^, activity, alertness, relaxation, and low nervousness^14^.

When interpreting the results we obtained, it is worth noting that, in general, in the entire sample of women coming from two relatively distant regions of Poland, the level of cortisol was very low, and the level of perceived stress was high. This may be related to women in Poland experiencing chronic stress in the early stages of pregnancy. Previous studies showed the relationships between cumulative risk exposure to stress and low cortisol level in adults^16,17,23^. The high levels of perceived stress and low hair cortisol concentration in Polish women in early pregnancy may be largely related to the very restrictive abortion law in force at the time of the tests and to the cases of several pregnant women dying in hospital due to doctors postponing the termination of a pregnancy that threatened the woman’s life. The deaths of these women were widely reported in the media during the period in which our study was conducted. The emotional state of pregnant women in Poland may also be related to difficulties with access to free health care and free prenatal tests.

Hypothesis 2, regarding time perspective as a mediator of the relationship between cortisol level and depressive symptoms, was partially confirmed. The results suggested that fatalistic time perspective explained the relationship between hair cortisol concentration in the first trimester and postpartum depressive symptoms. These results are consistent with research findings indicating the association of low cortisol levels with the perception of a difficult situation as insurmountable, a sense of helplessness, and exhaustion^18,19,23,45^, and also with the results suggesting the associations of high cortisol levels with the use of active coping strategies in challenging situations^11,14^.

As far as the limitations of our study are concerned, it should be noted that, although we controlled for many covariates, it was not possible to include all variables that may have influenced the results. For example, we did not ask about social support and whether the pregnancy was wanted or not. Another weakness of the study is the fact that we did not investigate the occurrence of any stressful events during pregnancy. Moreover, future research will need to examine the associations of very low maternal hair cortisol concentration during pregnancy with the course of pregnancy and fetal health. Many studies indicate the harmful effects of high maternal cortisol level on the child^47–49^, but there is a lack of research on the consequences of maternal hypocortisolism during pregnancy. This type of research is particularly important because too low cortisol levels may lead to a weakening of the body’s immune defense, which is very harmful to health^23^. It is worth referring here to studies conducted among Holocaust survivors and among their descendants. Both war victims and their children were characterized by low cortisol levels. However, in contrast to their parents, the children had elevated levels of the cortisol-reducing hormone. Scientists explain this phenomenon by an adaptive mechanism in the uterus. The hormone that breaks down cortisol is present in large amounts in the placenta, because its function is to protect the fetus from the mother’s cortisol. In war victims, both cortisol and cortisol-reducing hormone levels were low, which is probably why the placenta of mothers who survived the war had low levels of the cortisol-reducing hormone. For this reason, large amounts of cortisol passed into the fetus, which in turn produced large amounts of cortisol-reducing hormone. Low levels of cortisol and a high level of the enzyme that breaks it down in the bodies of children of war victims make them less adapted to life in their environment than their peers. This explains the fact that descendants of war survivors are more likely to develop post-traumatic stress disorder^50^. It would therefore be worthwhile to investigate in future studies whether children of mothers with low cortisol levels in early pregnancy are more susceptible to the negative consequences of stress.

Moreover, in further studies it would be worth checking whether the generally low hair cortisol level is characteristic only of women in the first weeks of pregnancy in Poland or, perhaps, culture-specific. This direction of research is all the more justified as researchers point to the influence of cultural factors on the general level of cortisol in the population^22^. Perhaps Polish people’s cortisol level is influenced by their frequently fatalistic attitude towards time, the belief that life is ruled by fate and there is little that one can change in one’s own future^51,52^. It is also worth noting that the mental state of women participating in our study could have been influenced by the experience of the COVID 19 pandemic. The high level of perceived stress could be related to concerns about the danger of infection and related pregnancy complications, as well as negative post-COVID effects see^53,54^. It should also be added that we measured hair cortisol concentration using only one of several methods currently used see^55^. It would be worthwhile to repeat the measurements using other techniques for analyzing hair cortisol concentration.

It was very interesting that the occurrence of postpartum depression in the mother or sister of a woman correlated positively with her level of pregnancy depression, but was not associated with her level of postpartum depression symptoms. Perhaps when a woman is pregnant, she is more influenced by family messages and observations of her closest relatives regarding coping with motherhood. However, when the woman herself has given birth and sees that she is coping well, the power of her relatives’ negative experiences no longer affects her to such a large extent. It is worth verifying these results in further studies.

Finally, it is worth mentioning that the results of some studies indicate differences in hair cortisol levels in women in their first and subsequent pregnancies^56^. In our study, there were no significant differences between primipara and multipara, in terms of stress, cortisol and depression. However it would be worth investigating possible differences in a larger group of subjects.

To sum up, the results of our study indicate that women with very low hair cortisol concentration in the first trimester are at greater risk for postpartum depression. These are the first data to show that low level of cortisol in the first trimester can be a predictor of maternal postpartum depressive symptoms. The results of our study suggest that examining hair samples in the first trimester of pregnancy may be a valuable method for identifying women at risk of developing postpartum depression.

## Supplementary Information

Supplementary Information.

## Data Availability

The data that support the findings of this study are openly available in OSF at https://osf.io/w4cgb/

## Abbreviations

EPDS: The Edinburgh Postnatal Depression Scale
PSS-10: The Perceived Stress Scale
ZTPI: The Zimbardo Time Perspective Inventory
PF: Present Fatalistic scale
PN: Past Negative scale
HPA: The hypothalamic–pituitary–adrenal
HCC I: Hair cortisol concentration in the first trimester
