# Supplementary material for: The effect of cortisol in early pregnancy on postpartum depressive symptoms
Source: Sci Rep. 2025 Apr 16;15:13108. doi: 10.1038/s41598-025-88772-0 (PMC12003845; doi:10.1038/s41598-025-88772-0)
Supplement: Supplementary file 1 — Supplementary Information. [file 41598_2025_88772_MOESM1_ESM.doc]

**Appendix**

Correlations of clinical characteristics with depressive symptoms, perceived stress, time perspective and hair cortisol concentration in the first trimester and postpartum

|  | EPDS I | PSS-10 I | PF I | PN I | HCC I | EPDS II | PSS-10 II |  |  |
| --- | --- | --- | --- | --- | --- | --- | --- | --- | --- |
| 1. Miscarriages in the past | .03 | -.09 | -.15 | -.09 | -.05 | -.05 | .11 |  |  |
| 2. History of mood disorders | -.02 | .12 | -.19 | -.07 | .05 | .04 | -.02 |  |  |
| 3. Cases of postpartum depression in mother or sister | .20* | .20* | .04 | .10 | .02 | .05 | .02 |  |  |
| 4.The presence of a chronic disease | .10 | .12 | -.09 | .12 | .08 | .09 | .24* |  |  |
| 5. Normal fetal development in the first trimester | .13 | .05 | .10 | .09 | .11 | .13 | .11 |  |  |
| 6. Symptoms of threatened miscarriage in the first trimester | .09 | -.09 | -.12 | -.07 | .09 | -.10 | .01 |  |  |
| 7. Blood glucose level in the first trimester < 92 | .09 | .04 | .06 | .07 | .05 | .04 | .13 |  |  |
| 8. Blood thyrotropic hormone level in the first trimester < 2.5 | .09 | .12 | .06 | .10 | .01 | .15 | .02 |  |  |
| 9. Premature birth (< 37 week) | .01 | .10 | .08 | .10 | .05 | .11 | .03 |  |  |
| 10. Caesarean section |  |  |  |  |  |  |  |  |  |

Note. *p < .05; **p < .01; ***p < .001; HCC I = hair cortisol concentration in the first trimester; EPDS I = Edinburgh Postnatal Depression Scale in the first trimester; PSS-10 I = Perceived Stress Scale in the first trimester; PF I = Present Fatalistic scale in the first trimester; PN I = Past Negative scale in the first trimester; EPDS II = Edinburgh Postnatal Depression Scale during postpartum; PSS-10 II = Perceived Stress Scale during postpartum
